# Prenatal anxiety, tobacco use, and infant birth weight: a multilevel analysis of PRAMS data

**DOI:** 10.1007/s00737-026-01773-4

**Published:** 2026-09-26

**Authors:** Sunghyun Chung, Lawrence Watkins

**Affiliations:** Department of Health Behavior, Texas A&M School of Public Health, Texas A&M University, College Station, USA

**Keywords:** Maternal anxiety, Prenatal tobacco use, Infant birth weight, Multilevel modeling, PRAMS

## Abstract

**Purpose:**

Maternal anxiety is a clinically relevant mental health concern during pregnancy and may contribute to reduced fetal growth, particularly when co-occurring with prenatal tobacco use. To examine the individual and contextual associations of maternal anxiety and prenatal tobacco use with infant birth weight using 2021–2023 Pregnancy Risk Assessment Monitoring System data. The analytic sample included 29,288 mothers nested within 11 states or jurisdictions.

**Methods:**

Two-level linear mixed models were estimated to predict continuous infant birth weight, with a random slope for maternal anxiety to assess whether the anxiety–birth weight association varied across states or jurisdictions.

**Results:**

Prenatal tobacco use was associated with an approximately 219-gram reduction in birth weight (*p* < .001), while maternal anxiety was associated with an approximately 60-gram reduction (*p* < .001). State-level prevalence of anxiety and tobacco use did not directly predict birth weight; however, the association between maternal anxiety and birth weight varied across states or jurisdictions.

**Conclusions:**

These findings support integrated prenatal care approaches that combine maternal mental health screening, tobacco cessation support, and attention to contextual systems that may shape maternal and infant health.

## Article Highlights

- Prenatal tobacco use was associated with substantially lower infant birth weight.

- Maternal anxiety was independently associated with lower infant birth weight.

- The anxiety–birth weight association varied across states or jurisdictions in U.S.

- Findings support integrated prenatal mental health and tobacco cessation care.

## Introduction

Pregnancy is a critical period in which maternal mental health may influence both maternal functioning and fetal development. Anxiety during pregnancy is clinically important because it may affect health behaviors, stress physiology, prenatal care engagement, and birth outcomes (Field 2017). Infant birth weight is one measurable indicator of fetal growth and is associated with neonatal morbidity, mortality, and long-term developmental risk (Hansen et al. 2018). Prenatal tobacco exposure has been linked to lower infant birth weight and increased risk of low birth weight, commonly defined as birth weight below 2500 g (Havard et al. 2022). In the United States, the prevalence of low birth weight has fluctuated around 8.3% to 8.5% in recent years, indicating a persistent public health issue that requires further exploration (Delcroix et al. 2023; Schechter et al. 2018). Smoking during pregnancy is a modifiable risk factor recognized for increasing the incidence of LBW. Mechanistically, tobacco consumption can impair fetal growth through compromised placental function, resulting in lower birth weights and subsequent health ramifications (Nadhiroh et al. 2020; Shrestha et al. 2022).

However, individual health behaviors do not occur in isolation. They are often shaped by the broader social and geographic contexts in which individuals live which can have a collective influence on health outcomes. State-level differences in healthcare access, social safety-net policies, tobacco control environments, socioeconomic conditions, and availability of prenatal health services (Crear-Perry et al. 2021; McCullough & Leider, 2017; Montez and Grumbach 2023). However, fewer studies have used multilevel approaches to distinguish individual-level associations from broader state-level contextual patterns in the relationship between maternal mental health, tobacco use, and infant birth weight.

Maternal psychological distress, particularly anxiety, may intensify the effects of smoking on fetal development (Arabzadeh et al. 2024). Elevated levels of perceived stress have been positively correlated with increased smoking rates and adverse birth outcomes, including LBW (Pereira et al. 2020; Yamamoto et al. 2023). The interaction between maternal anxiety and smoking behaviors during pregnancy can create a cyclical pattern where stress leads to smoking, which in turn heightens anxiety regarding potential adverse outcomes for the fetus (Suparno et al. 2021). The relationship between increased maternal anxiety and the likelihood of smoking, along with poor infant health, is underscored by several studies, highlighting the necessity for integrated support strategies for pregnant women facing these intertwined challenges (Dowse et al. 2020; Ghimire et al. 2021).

Moreover, some studies suggest that women may resort to smoking as a coping mechanism for anxiety, thus placing their unborn child at heightened risk for LBW (Ghimire et al. 2021; Gelaye et al. 2020). Prenatal stress has also been documented as a potential contributor to LBW, independent of substance use, making it essential to consider maternal mental health within reproductive health policies (Pereira et al. 2020; Grigoriadis et al. 2018; Shiferaw et al. 2018). Further exploration of these relationships will enhance our understanding of the etiology of low birth weight and inform intervention strategies targeting at-risk populations. Therefore, this study used a multilevel modeling approach to examine four questions: whether infant birth weight varied across states or jurisdictions; whether individual-level maternal anxiety and prenatal tobacco use were associated with infant birth weight; whether state-level prevalence of anxiety and tobacco use explained contextual differences in birth weight; and whether the association between maternal anxiety and infant birth weight varied across states or jurisdictions.

## Materials and methods

### Data source and participants

This study used data from the 2021–2023 Pregnancy Risk Assessment Monitoring System (PRAMS), a population-based surveillance system that collects information on maternal experiences before, during, and shortly after pregnancy. PRAMS links maternal survey responses with selected birth certificate information, allowing maternal health indicators to be examined in relation to infant birth outcomes.

The initial dataset included 59,343 observations. The analytic sample was constructed based on individuals with complete data on all model variables. We excluded individuals with missing data on the outcome variable (Birth Weight), individual-level predictors (Maternal Anxiety, Tobacco Use), or the state identifier. The final analytic sample consisted of 29,288 mothers/respondents (Level 1) nested within 11 states or jurisdictions (Level 2): Delaware (DE), Kansas (KS), Michigan (MI), Missouri (MO), Montana (MT), New Jersey (NJ), Pennsylvania (PA), Utah (UT), Washington (WA), Wisconsin (WI), and New York City (YC).

### Measures

#### Infant birth weight

Infant birth weight was obtained from birth certificate data and analyzed as a continuous measure in grams. In the PRAMS analytic file, birth weight was categorized in 250-gram intervals ranging from 125 g to 6375 g. The midpoint of each 250-gram category was used to approximate continuous birth weight in grams for the multilevel models.

#### Maternal anxiety

Maternal anxiety was measured using a self-reported item indicating whether the respondent had been told by a healthcare provider that she had anxiety before or during pregnancy. Responses were coded as 1 = yes and 0 = no.

#### Prenatal tobacco use

Prenatal tobacco use was measured using a self-reported indicator of tobacco use during pregnancy. Responses were coded as 1 = yes and 0 = no.

#### State-level context

State-level anxiety prevalence and state-level tobacco-use prevalence were calculated by aggregating individual-level maternal anxiety and prenatal tobacco-use indicators within each state or jurisdiction. These aggregate variables were used to examine whether contextual prevalence of anxiety or tobacco use was associated with infant birth weight beyond individual-level anxiety and tobacco use.

### Statistical Analysis

Analyses were restricted to the 11 states or jurisdictions that provided complete data on infant birth weight, maternal anxiety, prenatal tobacco use, and state identifier. Descriptive statistics were used to summarize individual- and state-level characteristics. All analyses were conducted using Stata/SE 18.0 (StataCorp, College Station, TX).

Two-level linear mixed models were estimated using Stata’s mixed procedure (StataCorp 2023) with mothers/respondents nested within states or jurisdictions. To distinguish individual-level from state-level associations, maternal anxiety and prenatal tobacco use were centered within state or jurisdiction, and state-level anxiety and tobacco-use prevalence variables were grand-mean centered. The state-level prevalence variables were rescaled so that coefficients represented a 10-percentage-point difference in prevalence.

An unconditional random-intercept model was first estimated to assess between-state variation in infant birth weight and calculate the intraclass correlation coefficient. A full conditional model was then estimated with individual-level maternal anxiety and prenatal tobacco use, state-level anxiety and tobacco-use prevalence, and a random slope for maternal anxiety to assess whether the anxiety–birth weight association varied across states or jurisdictions. An unstructured covariance matrix was used to estimate the intercept variance, maternal anxiety slope variance, and their covariance. Model fit was compared using a likelihood ratio test. Post-estimation empirical Bayes estimates were calculated within Stata using the predict, reffects command (StataCorp 2023) to describe state- or jurisdiction-specific maternal anxiety slopes. Predictive margins were estimated to visualize adjusted differences in infant birth weight by prenatal tobacco use. Detailed model specifications are provided in Online Resource 1.

## Results

### Descriptive statistics

The final analytic sample consisted of 29,288 mothers/respondents nested within 11 states or jurisdictions. The mean infant birth weight for the analytic sample was 3,074 g (SD = 738). Regarding individual risk factors, 6.4% (*n* = 1,882) of mothers reported tobacco use during pregnancy, and 29.2% (*n* = 8,548) reported a diagnosis of anxiety. At the contextual level, the average state-level anxiety prevalence was 29.2% (SD = 7.6%), and the average state-level tobacco use prevalence was 6.7% (SD = 3.2%). Detailed descriptive statistics are presented in Table 1.

**Table 1:** Individual- and state-level sociodemographic and behavioral characteristics of the analytic sample (*N* = 29,288) Across 11 U.S. States or jurisdictions, PRAMS 2021–2023

Characteristic	*N* (%) or Mean (SD)
Level 1: Individual characteristics (*N* = 29,288)	
Infant birth weight (grams)	3,074.0 (738.0)
Maternal anxiety (Yes)	8,548 (29.19%)
Tobacco use (Yes)	1,882 (6.43%)
Level 2: State/jurisdiction characteristics (*N* = 11)	
State-Level anxiety prevalence	29% (7.6%)
State-Level tobacco prevalence	7% (3.2%)
State-Level sample distribution	N (%)
Delaware (DE)	1,910 (6.52%)
Kansas (KS)	2,880 (9.83%)
Michigan (MI)	3,337 (11.39%)
Missouri (MO)	2,121 (7.24%)
Montana (MT)	1,642 (5.61%)
New Jersey (NJ)	2,577 (8.80%)
Pennsylvania (PA)	2,750 (9.39%)
Utah (UT)	3,376 (11.53%)
Washington (WA)	2,975 (10.16%)
Wisconsin (WI)	2,508 (8.56%)
NYC (New York City)	3,212 (10.97%)

*Note: Level 1 statistics are N (%). Level 2 statistics are Mean (SD) of state-level prevalences.*

### Between-state variation in infant birth weight

The unconditional random-intercept model indicated between-state or between-jurisdiction variation in infant birth weight. The estimated random-intercept variance was 30,289.49, and the residual variance was 444,048.20. The intraclass correlation coefficient was 0.063, indicating that approximately 6.3% of the total variance in infant birth weight was attributable to differences between states or jurisdictions. This finding supported the use of a multilevel modeling approach to account for the nesting of mothers/respondents within states or jurisdictions.

### Individual-level predictors of infant birth weight

**Fig. 1:**
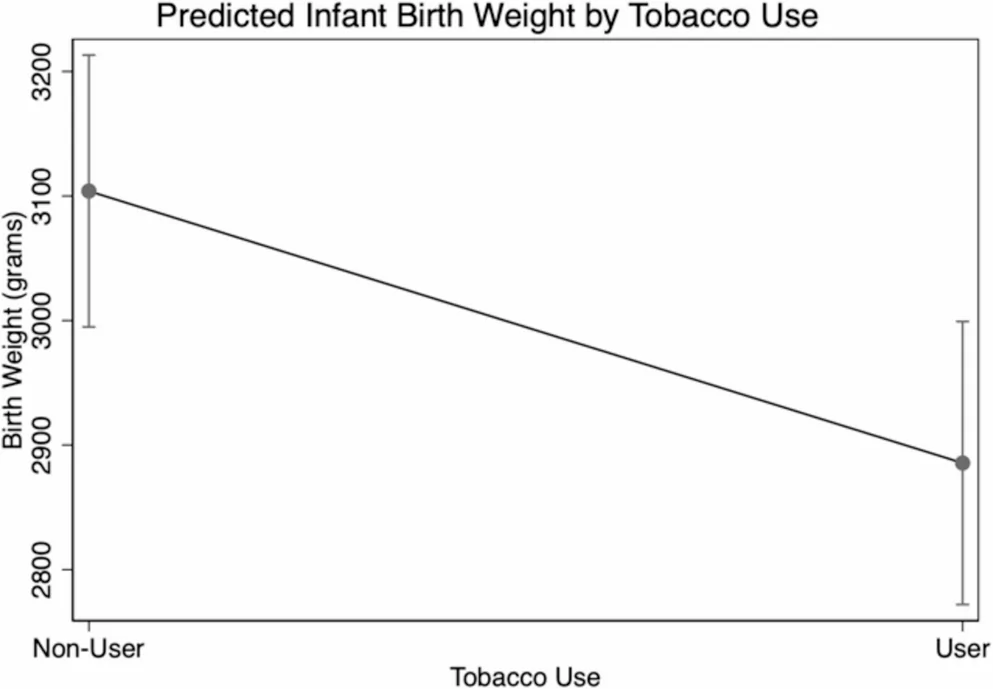
Adjusted predicted infant birth weight in grams by prenatal tobacco use status, accounting for maternal anxiety and state-level variation. Note. Points represent adjusted predicted margins from a multilevel model including prenatal tobacco use and maternal anxiety, with random intercepts for state or jurisdiction. Error bars indicate 95% confidence intervals. Although the confidence intervals for the predicted absolute means overlap due to the inclusion of broader model variance (e.g., state-level and residual variance), the specific within-state difference between tobacco users and non-users is estimated with high precision, yielding a highly significant main effect (p < .001).

In the full conditional model, both maternal anxiety and prenatal tobacco use were associated with lower infant birth weight. Maternal anxiety was associated with a 59.94-gram reduction in birth weight (b = -59.94, SE = 12.84, *p* < .001), while prenatal tobacco use was associated with a larger 218.72-gram reduction (b = -218.72, SE = 17.33, *p* < .001). Full estimates from the null and full multilevel models are presented in Table 2. Predictive margins from a multilevel model including prenatal tobacco use and maternal anxiety showed an adjusted mean birth weight of approximately 2,886 g among infants born to mothers who used tobacco during pregnancy, compared with approximately 3,104 g among infants born to mothers who did not use tobacco (Fig. 1). In the fully adjusted model (Model 3 in Table 2), which controlled for maternal sociodemographic characteristics (age, education, race/ethnicity, marital status, and household income) and prenatal e-cigarette use, both prenatal tobacco use and maternal anxiety remained robust and statistically significant predictors of lower infant birth weight. Specifically, prenatal tobacco use was associated with an adjusted 213.13-gram reduction in birth weight (b = -213.13, SE = 18.96, *p* < .001), and maternal anxiety was associated with an 81.57-gram reduction (b = -81.57, SE = 11.27, *p* < .001). Among the newly added controls, prenatal e-cigarette use, maternal age, race/ethnicity, and marital status also showed significant associations with birth weight, as detailed in Table 2. 

**Table 2:** Multilevel linear mixed models predicting infant birth weight in grams by maternal anxiety, prenatal tobacco use, and sociodemographic factors

Predictor	Model 1: Null Model	Model 2: Main Effects	Model 3: Fully Adjusted
	b (SE) [*p*-value]	b (SE) [*p*-value]	b (SE) [*p*-value]
Fixed Effects			
Intercept	3133.10 (36.41) [< 0.001]	3083.76 (51.40) [< 0.001]	3025.22 (74.42) [< 0.001]
Level 1: Main predictors			
Maternal anxiety	—	-59.92 (12.84) [< 0.001]	-81.57 (11.27) [< 0.001]
Prenatal tobacco use	—	-218.73 (17.33) [< 0.001]	-213.13 (18.96) [< 0.001]
Level 2: State-level predictors			
State anxiety prevalence^a	—	-742.53 (847.17) [0.381]	-1631.66 (917.27) [0.104]
State tobacco prevalence^a	—	2075.59 (2032.63) [0.307]	2671.38 (2197.43) [0.250]
Level 1: Sociodemographic & behavioral controls			
E-cigarette use (Ref: No)	—	—	137.65 (50.74) [0.007]
Maternal age (years)	—	—	-18.53 (4.09) [< 0.001]
Maternal education (years)	—	—	4.69 (5.10) [0.357]
Race/Ethnicity (Ref: Non-Hispanic white)^b			
Non-Hispanic Black	—	—	-199.28 (13.92) [< 0.001]
Hispanic	—	—	-58.26 (13.20) [< 0.001]
Non-Hispanic Asian/PI	—	—	-237.71 (15.81) [< 0.001]
Non-Hispanic AI/AN	—	—	119.31 (27.63) [< 0.001]
Mixed/Other Race	—	—	-43.53 (20.26) [0.032]
Marital Status (Ref: Married)			
Unmarried / other	—	—	-53.85 (10.94) [< 0.001]
Household Income (Ref: ≤ $20,000)^b			
$20,001 - $40,000	—	—	32.68 (14.84) [0.028]
$40,001 - $60,000	—	—	58.33 (16.62) [< 0.001]
≥ $60,001	—	—	72.95 (15.62) [< 0.001]
Missing / Unknown	—	—	22.44 (17.63) [0.203]
Random effects (Variance components)			
State-level intercept variance	30289.49 (8990.30)	28521.42 (13243.69)	26575.50 (163.02)^c
Maternal anxiety slope variance	—	813.06 (717.45)	389.90 (19.75)^c
Residual variance	444048.20 (2579.10)	506439.70 (4186.46)	496397.60 (704.55)^c
ICC	0.064	—	—

Note. *N* = 29,278 mothers nested within 11 states or jurisdictions for Model 3 (10 observations excluded due to missing e-cigarette data). Individual-level predictors were group-mean centered, and state-level prevalence variables were grand-mean centered. Exact *p*-values are provided for all statistical tests as recommended. ^a State-level coefficients represent differences based on grand-mean centering. ^b Reference groups represent the baseline categories from the PRAMS standard coding. Household Income data utilized a missing indicator approach to preserve sample size. ^c Variance components for Model 3 display variance estimates and standard deviations (SD) generated by the lme4 package

### State-level contextual predictors of infant birth weight

State-level anxiety prevalence and state-level tobacco-use prevalence were not statistically significant predictors of infant birth weight in the full conditional model. State-level anxiety prevalence was not significantly associated with infant birth weight when modeled per 10-percentage-point difference above the grand mean (b = -75.68, SE = 83.33, *p* = .364). Similarly, state-level tobacco-use prevalence was not significantly associated with infant birth weight (b = 211.39, SE = 199.61, *p* = .290).

### State-level variation in the anxiety–birth weight association

**Fig. 2:**
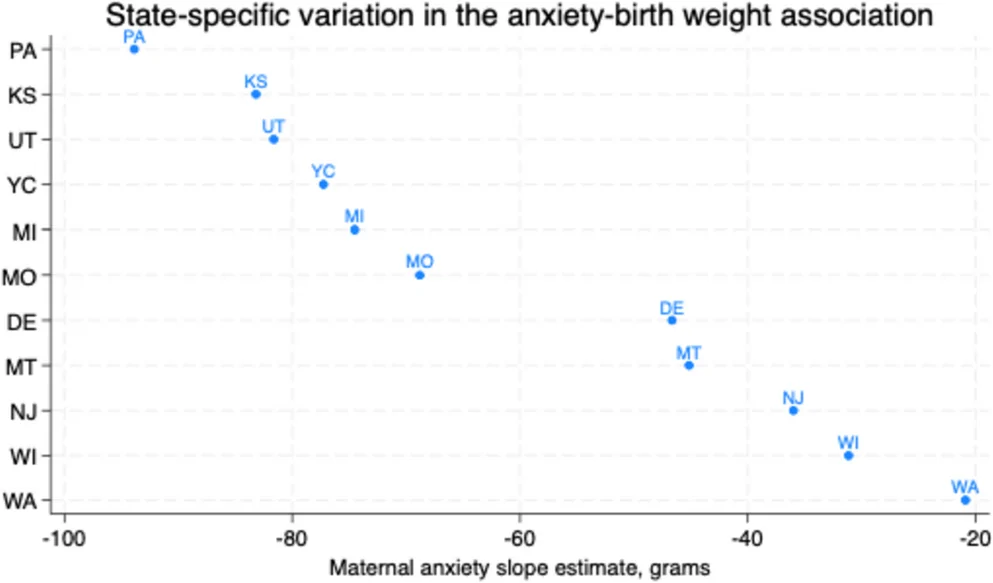
State- or jurisdiction-specific empirical Bayes estimates of the association between maternal anxiety and infant birth weight in grams. Note. State- or jurisdiction-specific slope estimates were calculated by adding the fixed effect for maternal anxiety to the empirical Bayes random slope for each state or jurisdiction. More negative estimates indicate a stronger association between maternal anxiety and lower predicted infant birth weight. Estimates are descriptive and should not be interpreted as formal pairwise comparisons between states or jurisdictions.

The random-slope model indicated significant state- or jurisdiction-level variation in the association between maternal anxiety and infant birth weight. The estimated variance component for the maternal anxiety slope was 813.69, with a 95% CI that did not include zero [144.40, 4585.02]. As shown in Fig. 2, post-estimation empirical Bayes estimates suggested that the negative anxiety–birth weight association was strongest in Pennsylvania, Kansas, and Utah and weakest in Washington, Wisconsin, and New Jersey. These descriptive estimates should not be interpreted as formal state-to-state comparisons; detailed state- or jurisdiction-specific estimates are provided in Online Resource 2. 

## Discussion

### Principal findings

This study examined maternal anxiety and prenatal tobacco use as mental health-related and behavioral predictors of infant birth weight using multilevel PRAMS data from 29,288 mothers/respondents across 11 states or jurisdictions. Three principal findings emerged. First, prenatal tobacco use was associated with substantially lower infant birth weight. Second, maternal anxiety was independently associated with lower infant birth weight, although the magnitude of this association was smaller than that observed for prenatal tobacco use. Third, the association between maternal anxiety and infant birth weight varied across states or jurisdictions, suggesting that the relationship between maternal psychological distress and fetal growth may not be uniform across contextual settings.

In contrast, state-level prevalence of anxiety and tobacco use was not directly associated with infant birth weight in the full conditional model. This finding should be interpreted cautiously because the analysis included only 11 Level-2 units, which may have limited statistical power to detect state-level contextual effects. Overall, the findings suggest that individual-level maternal anxiety and prenatal tobacco use were more consistently associated with infant birth weight than the aggregate state-level prevalence indicators included in this analysis.

### Maternal anxiety, prenatal tobacco use, and infant birth weight

The finding that prenatal tobacco use was associated with lower infant birth weight is consistent with prior evidence linking tobacco exposure during pregnancy to adverse birth outcomes (Pereira et al. 2017; Di et al. 2022). In this study, prenatal tobacco use was associated with an adjusted reduction of approximately 219 g in infant birth weight. This magnitude is meaningful because even moderate reductions in birth weight may increase vulnerability to neonatal complications and longer-term developmental risks (Amadou et al. 2025). These results reinforce the importance of tobacco cessation support as a central component of prenatal care. Given the evolving landscape of nicotine use, we additionally adjusted the fully adjusted model for prenatal e-cigarette use to account for potential confounding by concurrent vaping behavior (Ammar et al. 2023). The inverse associations of prenatal combustible tobacco use and maternal anxiety with infant birth weight remained after this adjustment, suggesting that these associations were not explained by e-cigarette use. This finding underscores the importance of distinguishing combustible tobacco use from other forms of prenatal nicotine exposure while recognizing the complexity of contemporary nicotine-use patterns during pregnancy (Regan et al. 2021).

Maternal anxiety was also associated with lower infant birth weight, with an adjusted reduction of approximately 60 g. Although this association was smaller than the association observed for prenatal tobacco use, it remained statistically significant after accounting for tobacco use and state-level contextual indicators. This finding supports the broader literature suggesting that maternal psychological distress during pregnancy may be linked to fetal growth outcomes through behavioral, physiological, and healthcare-related pathways (Araji et al. 2020; Grigoriadis et al. 2018). Anxiety may serve as a contributor to maladaptive coping behaviors or other behavioral risk factors, including tobacco use, which represent a more proximal determinant of adverse perinatal outcomes such as low birth weight.

Moreover, maternal anxiety and prenatal tobacco use should not be viewed as entirely separate risk factors. Pregnant women frequently smoke in the presence of significant psychological distressors, suggesting that tobacco use may serve a coping function during pregnancy (Sequí-Canet et al. 2022). While our findings demonstrate that maternal anxiety and tobacco use independently predict reduced birth weight, this independence at the statistical level does not rule out a behavioral link in which women use tobacco to manage anxiety. These observations point to maternal mental health as a meaningful intervention target and underscore the need for integrated prenatal care approaches that address psychological distress and tobacco use jointly rather than as isolated clinical concerns.

Furthermore, our fully adjusted model highlights the critical importance of sociodemographic context. Previous research clearly demonstrates that maternal smoking rates and prenatal tobacco exposure are not uniform across racial and ethnic groups (Hoshiko et al. 2019). By incorporating race and ethnicity into our final model, we accounted for these known disparities. The finding that tobacco use and anxiety remained significant predictors of low birth weight, even after controlling for race and ethnicity, underscores the robust nature of these individual-level risk factors across diverse population groups.

### State-level variation in the anxiety–birth weight association

A notable finding of this study was the significant random slope for maternal anxiety, indicating that the association between maternal anxiety and infant birth weight varied across states or jurisdictions. This suggests that the fetal growth implications of maternal anxiety may depend partly on broader contextual conditions. Although the present analysis did not directly test specific state-level policy or healthcare factors as moderators, the observed variation may reflect unmeasured differences in prenatal care access, availability of maternal mental health services, socioeconomic conditions, tobacco control environments, or social support infrastructure which other literature has shown to directly influence maternal health outcomes (Chang et al. 2024). Post-estimation empirical Bayes estimates further clarified this heterogeneity. The negative association between maternal anxiety and infant birth weight appeared strongest in Pennsylvania, Kansas, and Utah and weakest in Washington, Wisconsin, and New Jersey. Because these estimates were derived from a random-slope model with only 11 Level-2 units, they should be interpreted as descriptive evidence of heterogeneity rather than as definitive evidence that specific state policy or healthcare environments caused stronger or weaker associations.

To further illustrate the importance of state-level factors, results on national data indicate that maternal mental health resources are unevenly distributed, with the South and rural regions consistently demonstrating fewer perinatal health workers, higher rates of postpartum depression symptoms, and greater concentrations of maternity care deserts (United States of Care, 2025). Approximately 65% of rural U.S. counties lack a practicing psychiatrist, and the majority of rural residents live in federally designated mental health professional shortage areas (Health Resources and Services Administration 2024). In states where prenatal mental health resources are scarce, anxiety may exert a stronger association with low birth weight precisely because symptoms are less likely to be identified, treated, or buffered by formal and informal supports suggesting that individual anxiety carries different downstream risk depending on the structural context in which a pregnancy takes place (Kozhimannil et al. 2024).

At the same time, the non-significant fixed effects for state-level anxiety and tobacco-use prevalence suggest that aggregate prevalence indicators may not adequately capture the structural and policy-level conditions that shape perinatal outcomes. Prevalence reflects population-level burden, but it does not directly measure the systems that determine whether that burden translates into adverse outcomes. Evidence from natural experiments examining Medicaid expansion, for example, suggests heterogeneous effects on low birth weight that vary substantially by state context and racial/ethnic subgroup, underscoring that policy environments (Brown et al. 2019). For example, because our analysis was limited to the 11 states with complete tobacco use data, we could not examine anxiety or tobacco use prevalence in southern states, where higher rates of tobacco use are well documented (Patel et al. 2026). Incorporating such direct contextual measures in subsequent multilevel analyses may help clarify why the anxiety–birth weight association appears stronger in some states or jurisdictions than in others and may move the field beyond descriptive variation toward actionable, policy-relevant explanation.

It is also imperative to contextualize these findings within the timeframe of the data collection (2021–2023), which coincided with the protracted phases of the COVID-19 pandemic. The pandemic drastically altered the complex interactions between community-level infrastructure and individual health behaviors. Disruptions to prenatal care access, social isolation, and heightened systemic uncertainty likely exacerbated maternal psychological distress while simultaneously altering coping mechanisms, such as tobacco use. Consequently, the state-level variations observed in the anxiety–birth weight association may partly reflect differences in state-level pandemic responses, the resilience of local healthcare safety nets, and the varying degrees of disruption to maternal mental health and cessation services during this unprecedented period. Future studies should continue to investigate how these pandemic-era behavioral shifts have long-term implications for maternal and infant health trajectories.

### Implications for prenatal mental health and tobacco cessation

The findings support an integrated approach to prenatal care that combines mental health screening with tobacco cessation support. Because maternal anxiety and prenatal tobacco use were both associated with lower infant birth weight, clinical and public health programs should consider screening for these risks together. For example, pregnant individuals who report anxiety may benefit from additional assessment of tobacco use, coping behaviors, and access to mental health treatment. Similarly, individuals who report tobacco use during pregnancy may benefit from anxiety screening and referral to behavioral health services.

These findings are also relevant to psychiatric and psychological practice because they highlight pregnancy as a period in which mental health symptoms may have implications beyond maternal well-being alone. Maternal anxiety may be associated with fetal growth outcomes, particularly when combined with behavioral risk factors such as tobacco use. Therefore, prenatal mental health care should be framed not only as symptom management but also as part of a broader strategy to support maternal and infant health. At the policy level, the observed variation across states or jurisdictions suggests that improving birth outcomes may require both individual-level and contextual strategies. Individual-level interventions, such as smoking cessation counseling and anxiety screening, remain essential. However, these approaches may be more effective when supported by broader systems that improve access to prenatal mental health services, reduce barriers to behavioral healthcare, and strengthen social support for pregnant individuals.

### Limitations

This study has several limitations. PRAMS includes self-reported measures, which may be affected by recall or social desirability bias, particularly for prenatal tobacco use. Maternal anxiety was based on a self-reported provider diagnosis rather than a validated symptom severity scale, and tobacco use was measured as a binary indicator without information on timing, intensity, cessation, or secondhand smoke exposure. The observational design limits causal inference, and several potential confounders, including demographic, clinical, and prenatal care factors, were not included. In addition, the analysis included only 11 states or jurisdictions, which may limit power for state-level effects and affect the stability of random-slope estimates. Finally, aggregate state-level prevalence measures may not fully capture broader policy or healthcare contexts, and the findings should not be interpreted as nationally representative.

## Conclusions

This study found that maternal anxiety and prenatal tobacco use were associated with lower infant birth weight in a multilevel analysis of PRAMS data from 11 states or jurisdictions. Prenatal tobacco use showed the larger association, corresponding to an adjusted reduction of approximately 219 g, while maternal anxiety showed a smaller but statistically significant association, corresponding to an adjusted reduction of approximately 60 g. The observed variation in the association between maternal anxiety and infant birth weight across states or jurisdictions suggests that the fetal growth implications of maternal psychological distress may not be uniform across contexts. These findings support integrated prenatal care approaches that combine maternal mental health screening, tobacco cessation support, and attention to broader contextual conditions that may shape maternal and infant health. Future research should examine whether specific policy, healthcare access, and social support indicators explain state- or jurisdiction-level variation in the association between maternal anxiety and infant birth weight.

## Data Availability

The data used in this study were obtained from the Pregnancy Risk Assessment Monitoring System (PRAMS), administered by the Centers for Disease Control and Prevention. Restrictions apply to the availability of these data. PRAMS data are not publicly available in the form used for this analysis and may be accessed from the CDC subject to PRAMS data access requirements and approval.
